# Percutaneous left atrial appendage closure using a modified single-operator-technician approach under deep sedation: A single-center experience

**DOI:** 10.1016/j.hroo.2024.10.004

**Published:** 2024-10-21

**Authors:** Alejandro José Quiroz Alfaro, Noah E. Russell, Ruhul Munshi, Waleed Hassan, James E. Stone, Elsheikh M. Abdelrahim, Karl J. Crossen, Karthik Venkatesh Prasad

**Affiliations:** 1 Department of Internal Medicine, North Mississippi Medical Center, Tupelo, Mississippi; 2 Department of Electrophysiology, North Mississippi Medical Center, Tupelo, Mississippi

**Keywords:** Atrial appendage closure, Deep sedation, General anesthesia, Atrial fibrillation, Electrophysiology, Same-day discharge, Left atrial appendage, Stroke prevention, Transesophageal echocardiography, Long-term outcomes

## Abstract

**Background:**

Historically, percutaneous transcatheter left atrial appendage closure (LAAC) has been performed under general anesthesia (GA) with transesophageal echocardiographic images obtained by a noninvasive cardiologist and usually requires an overnight hospital stay. Alternatively, we present our single-center experience performing LAACs under deep sedation (DS), employing an echocardiographic technician instead of a noninvasive cardiologist, and expediting same-day discharge. Mid- to long-term outcomes were also evaluated with follow-up imaging at a 45-day visit.

**Objective:**

The purpose of this study was to demonstrate the safety, feasibility, and outcomes of our single-operator-technician LAAC approach.

**Methods:**

A total of 150 patients, with elevated CHA_2_DS_2_-VASc scores (a mean of 4 points), underwent transesophageal echocardiography–guided LAAC using the WATCHMAN FLX (Boston Scientific, Marlborough, MA) device under DS.

**Results:**

The mean age of patients was 78 years. Seventy-six (51%) were men. One hundred forty-seven patients (98%) had the LAAC device successfully implanted, and 145 (97%) were discharged on the same day. Nine patients (6%) required conversion from DS to GA. Only 5 patients (4%) had complications during the procedure. None of the patients died or had complications from DS. During the 45-day follow-up visit, one patient had a significant peridevice leak (maximum diameter ≥ 5 mm) and another patient had device-related thrombosis.

**Conclusion:**

Our novel single-operator-technician approach under DS is safe and feasible. Implementing protocols to simplify the traditional 2-operator approach under GA by using DS and an echocardiography technician as well as incorporating same-day discharge could make LAACs more widely available and potentially reduce procedural costs.

**Key Findings**

▪ Performing percutaneous left atrial appendage closures with an approach involving same-day preoperative imaging and postoperative discharge could reduce costs and recovery time and increase the availability of the procedure to more medical centers.
▪ Deep sedation can be a safe alternative to general anesthesia when performing percutaneous left atrial appendage closures, potentially reducing the average hospital length of stay.
▪ As demonstrated, a properly trained technician can safely replace the role of a noninvasive cardiologist and operate the transesophageal echocardiography probe under the guidance and supervision of the implanting physician, with an implant success rate comparable to the one reported nationwide between August 2020 and September 2022 in the United States.

## Introduction

Atrial fibrillation (AF) is the most common cardiac dysrhythmia, and its incidence and prevalence are increasing globally. Despite our understanding of modifiable risk factors associated with AF and its significant impact on patients' lives and health care systems, there is still a lack of programs aimed at preventing AF.^1^ Hence, it is projected that the prevalence of AF in the United States will substantially increase from ∼2.7 to 6.1 million in 2010 to an estimated 12.1 million patients by 2030.^2^

Because of its association with a 5-fold increase in stroke risk and the tendency for AF-related strokes to have a worse prognosis and greater severity, anticoagulation is commonly used to mitigate the risk of systemic embolization and ischemic strokes in patients with AF.^3^ As up to 91% of left atrial thrombi originate within the left atrial appendage (LAA), the surgical excision of the appendage was initially documented in cardiac surgery patients with rheumatic mitral valve stenosis and as a prophylactic measure against recurrent atrial thrombi.^4^

Subsequently, percutaneous transcatheter left atrial appendage closure (LAAC) emerged as a less invasive, nonpharmacological approach to mitigate the risk of thromboembolic events in nonvalvular AF, particularly in patients with an elevated thromboembolic risk, a history of significant bleeding, an increased risk of falls, or intolerance to anticoagulation.^4^, ^5^, ^6^

No consensus exists on the safest and most effective strategy to perform percutaneous transcatheter LAACs. Although general anesthesia (GA) has been historically preferred over deep sedation (DS) when performing LAACs, there is insufficient evidence assessing the impact of either type of anesthesia on the safety and outcomes of the procedure.^7^ Therefore, we present our single-center experience involving 150 patients undergoing LAAC using the WATCHMAN FLX (Boston Scientific, Marlborough, MA) appendage closure device under DS.

We implemented a novel approach using a single operator, the electrophysiologist, guiding an echocardiography technician who is registered and certified as a diagnostic cardiac sonographer by the American Registry for Diagnostic Medical Sonography or Cardiovascular Credentialing International instead of the conventional 2-operator (the interventionist and the noninvasive cardiologist) system under GA used by most centers. Mid- to long-term outcomes were also evaluated at a 45-day follow-up visit with follow-up imaging. We hypothesize that our approach under DS is safe, even in elderly patients with comorbid conditions.

## Methods

This single-center retrospective observational study gathered data through sampling from electronic medical records at North Mississippi Medical Center, Tupelo, MS, as well as information collected from the same patients through the National Cardiovascular Data Registry Left Atrial Appendage Occlusion Registry. The sources mentioned above identified all patients who underwent percutaneous transcatheter LAAC from September 1, 2021, to September 30, 2022.

Of the 220 patients admitted to the electrophysiology suite for percutaneous transcatheter LAAC between September 1, 2021, and September 30, 2022, a total of 150 fulfilled the inclusion criteria and were included in this study. The remaining 70 patients were excluded for the following reasons: the procedure was conducted from the beginning under GA; a closure device other than the WATCHMAN FLX device was used; or the procedure was terminated after it was determined that the appendage anatomy was unsuitable for percutaneous closure during preprocedural transesophageal echocardiography (TEE) before any intravascular access for the percutaneous procedure was obtained; or, if the patients met any exclusion criteria.

This study adheres to the Strengthening the Reporting of Observational Studies in Epidemiology guidelines. The study was conducted in strict adherence to the principles of the Helsinki Declaration. The institutional review board waived the requirement to oversee the study or obtain patient consent because the data were collected retrospectively, and all information obtained was anonymized.

### Study population

The WATCHMAN FLX device was implanted in patients who met the following criteria: They were ≥18 years of age, had nonvalvular atrial fibrillation, and required anticoagulation therapy for stroke prevention because of an elevated thromboembolic risk. However, these patients were either intolerant to anticoagulation, experienced significant bleeding from anticoagulation, or had an increased risk of falls.

**Figure 1:**
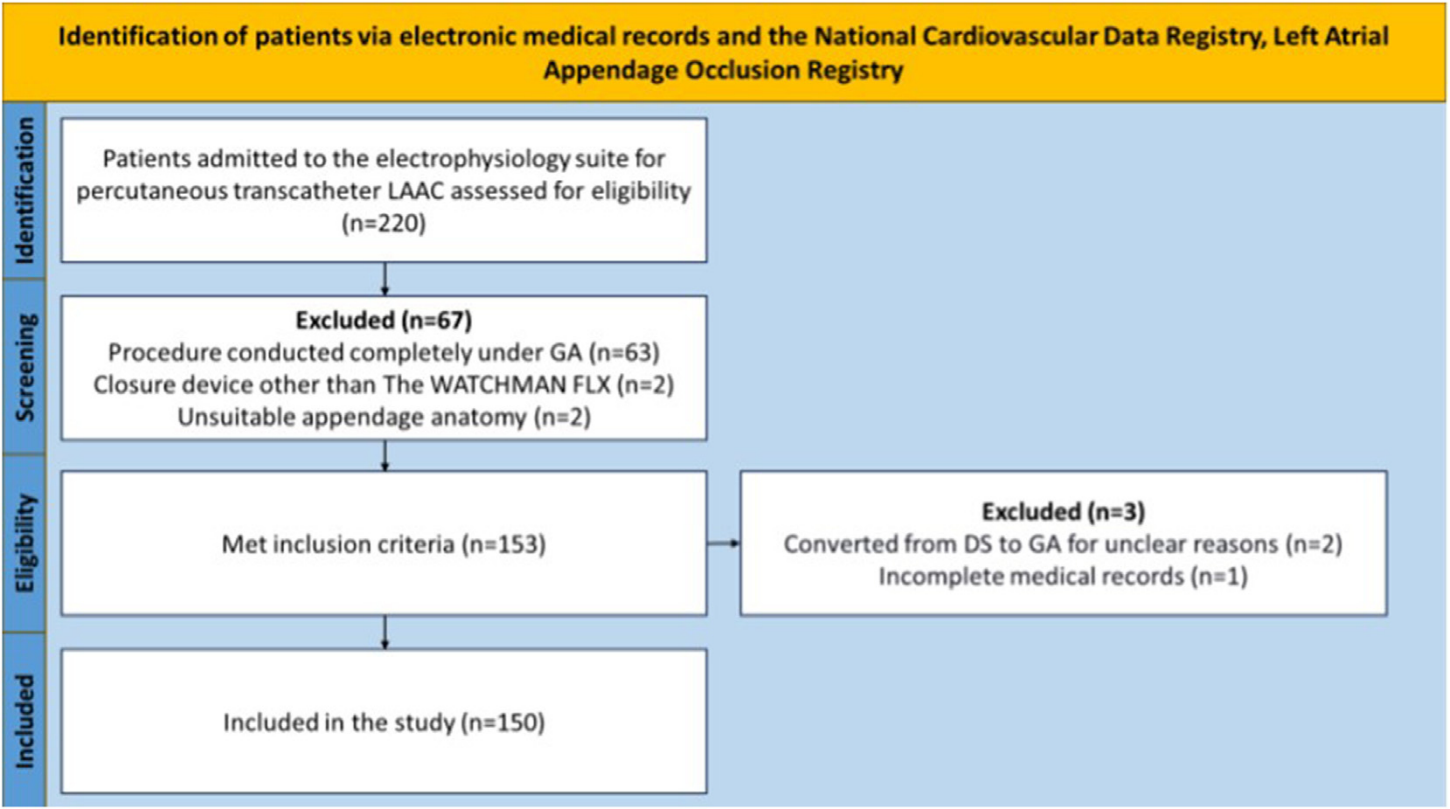
Strengthening the Reporting of Observational Studies in Epidemiology flowchart. DS = deep sedation; GA = general anesthesia; LAAC = left atrial appendage closure.

Inclusion criteria encompassed all patients 18 years or older who underwent percutaneous transcatheter LAAC under DS using the WATCHMAN FLX device between September 1, 2021, and September 30, 2022. This also included patients who required a conversion from DS to GA. Exclusion criteria included patients with incomplete or inconsistent medical records, or those who initially underwent LAAC under DS but lacked clear reasons for converting to GA (Figure 1).

The patients' baseline characteristics are listed in Table 1.

**Table 1:** Baseline characteristics (N = 150)

Variable	Value
Age (y)	78 ± 7
Sex	
Male	76 (51)
Female	74 (49)
BMI (kg/m^2^)	27 (24–31)
CHA_2_DS_2_-VASc score	4 (1)
Maximum ostial diameter (mm)	20.2 (18.4–22.2)
EF procedural TEE (%)	50 (45–55)

Values are presented as mean ± SD, median (interquartile range), or n (%).

BMI = body mass index; EF = ejection fraction; TEE = transesophageal echocardiography.

### Data extraction

The variables extracted from the medical records included age, sex, body mass index, CHA_2_DS_2_-VASc score, LAA maximum ostial diameter, left ventricular ejection fraction during preprocedural TEE, the type of anesthesia (DS), whether the patient was converted to GA, reasons for anesthesia conversion, discharge on the same day, and reasons for delayed discharge. During the procedure, variables assessed were the successful implantation of the device and the occurrence of acute complications such as pericardial effusion, pericardial effusion requiring intervention, ischemic stroke, and device dislodgment. At the 45-day follow-up visit, variables included TEE or cardiac computed tomography scan results, significant peridevice leak (PDL) (defined as maximum diameter ≥ 5 mm), and device-related thrombosis (DRT).

### Statistical analysis

All statistical analyses were performed using STATA 17 (StataCorp LLC, College Station, TX). Normality was initially assessed through histograms and the Shapiro-Wilk test for numerical variables. Data were described using means and standard deviations if a normal distribution was confirmed. Conversely, medians and interquartile ranges were used to represent numerical variables without a normal distribution. Categorical variables were presented using absolute and relative frequencies.

### Description of the procedure and follow-up

All patients underwent same-day TEE just before the LAAC procedure in the electrophysiology laboratory. This imaging study allowed us to accurately measure the appendage, abort the procedure if any thrombus within the appendage was found before any vascular access was obtained, and determine the adequate size for the closure device, minimizing complications. A certified cardiac sonographer operated the TEE probe at all times under the guidance and supervision of the electrophysiologist performing LAAC.

### DS protocol

Patients received supplemental oxygen via a face mask that would allow the TEE probe to pass. Sedation was administered intravenously by a certified registered nurse anesthetist or an anesthesiologist using intravenous propofol or propofol and midazolam, if necessary, to achieve and maintain a Richmond Agitation-Sedation Scale score of −2 to −4.

### Preprocedural TEE measurements

The maximum ostial diameter of the LAA was measured upon the insertion of the TEE probe. The LAA was evaluated at the level of the circumflex coronary artery, with the greatest measurement recorded as the maximum ostial diameter from the standard 4 angles at 0°, 45°, 90°, and 135°. This imaging study also allowed us to exclude the presence of a thrombus within the LAA. The left ventricular ejection fraction was calculated by visual estimation. Any baseline pericardial effusion was measured and documented. A complete study was performed to evaluate atrial septal defects, severe valvular dysfunction, or other contraindications for the percutaneous LAAC procedure.

### Device implantation

Under the guidance of TEE operated by the sonographer and fluoroscopy, the electrophysiologist would perform the WATCHMAN FLX device implantation using the technique described by Asmarats and Rodes-Cabau.^8^

After a successful and uncomplicated procedure, patients were transferred to the recovery room for around 45 minutes and then to an outpatient observation room, where they would be monitored for 4–5 hours with bed rest and discharged after the anticoagulation and anti-aggregation protocol on the same day.

In cases where complications arose during LAAC or the immediate postoperative period, patients were converted to GA and subsequently transferred to the intensive care unit, depending on the treatment requirements for the complications.

### Anticoagulation protocol after LAAC

After a successful LAAC procedure, patients were prescribed both an oral anticoagulant and single antiplatelet therapy, with options including aspirin, clopidogrel, or ticagrelor.

### 45-Day follow-up TEE

After 45 days, patients underwent follow-up TEE using the same angles (0°, 45°, 90°, and 135°). This follow-up aimed to assess the correct positioning of the occlusion device and to check for any PDL or DRT. The oral anticoagulant was discontinued if no DRT or PDL measuring ≥5 mm was detected. Subsequently, patients were advised to maintain dual antiplatelet therapy for a total of 6 months.

## Results

Of the 150 (100%) patients included in our study, 147 (98%) had the LAAC device successfully implanted, and 145 (97%) were discharged on the same day (Table 2). During the procedure, 9 (6%) patients required conversion from DS to GA (Table 3). Among patients requiring conversion, 7 had laryngospasms or difficulty passing the TEE probe, leading to oxygen desaturation.

**Table 2:** Implant success rate, complications, and follow-up (N = 150)

Variable	n (%)
Device successfully implanted	147 (98)
Complications during LAAC	
Pericardial effusion	5 (4)
Pericardial effusion requiring intervention	2 (2)
Ischemic stroke	0 (0)
Device dislodgment	0 (0)
Findings during 45-d follow-up imaging	
Leakage ≥ 5 mm	1 (<1)
Device thrombosis	1 (<1)
Same-day discharge	145 (97)

LAAC = left atrial appendage closure.

**Table 3:** Conversion to general anesthesia and follow-up complications (N = 150)

Variable	Anesthesia type		n (%)
	Deep sedation	Conversion to general anesthesia	
Complications during LAAC			
No complications	136	7	143 (96)
Pericardial effusion	3	2	5 (4)
Findings during 45-d follow-up imaging			
Device thrombosis	1	0	1 (<1)
Leakage ≥ 5 mm	1	0	1 (<1)
*Total*	*141*	*9*	*150 (100)*

LAAC = left atrial appendage closure.

Only 2 of the 9 were converted to GA because of procedural complications: one for a pericardial effusion resulting in tamponade from a right ventricular (RV) laceration and the other because of a pericardial effusion resulting from a perforation of the LAA during the implantation procedure.

Complications during the procedure were observed in 5 patients (4%): 3 (2%) had clinically insignificant pericardial effusions, 1 experienced pericardial tamponade owing to the RV laceration, and 1 had the effusion resulting from a perforation of the LAA. At the 45-day follow-up visit, during TEE, one patient had a PDL measuring ≥5 mm and another developed DRT.

## Discussion

Historically, the conventional LAAC approach involved several intricate components. Typically, preprocedural imaging was conducted on a separate day and interpreted by a specialist. The procedure itself required GA, and a noninvasive cardiologist would perform screening imaging during the procedure; then, an interventional cardiologist or electrophysiologist would implant the closure device. Even after an uneventful procedure, patients would usually undergo overnight hospital monitoring.

The conventional 2-operator approach under GA is inherently complex, demanding more time and a specialized multidisciplinary team. This complexity limits the widespread availability of LAAC procedures in some medical centers.^9^ Alternative approaches to perform LAAC have been developed to address this difficulty.^9^ These approaches aim to circumvent the need for GA by using protocols incorporating different levels of sedation, conducting same-day preprocedural imaging, and minimizing the number of operators required for the procedure.

Khan et al^10^ conducted a systematic review and meta-analysis to compare the safety, outcomes, and complications postdischarge between a same-day discharge strategy and hospital overnight admissions (>1 day) for patients undergoing percutaneous LAACs. The study found that there were no significant differences in all-cause mortality, ischemic stroke after discharge, readmissions up to 60 days after discharge, and PDL > 5 mm between the 2 groups.^10^ However, the same-day discharge group experienced significantly fewer major bleeding or vascular complications.^10^

Performing percutaneous LAACs with a same-day discharge strategy can potentially reduce up to 15% of the costs, improve patient satisfaction, and minimize the risk of hospital-acquired infections.^10^ Implementing a same-day discharge strategy may also increase the availability of percutaneous LAACs to more medical centers.

We achieved successful closure device implantation in 147 (98%) of our patients, which is comparable to the 97.5% successful implant rate reported nationwide in the National Cardiovascular Data Registry Left Atrial Appendage Occlusion Registry between August 2020 and September 2022.^11^ The 3 patients who did not receive the device had distinct reasons. One patient had unsuitable appendage anatomy for the closure device but encountered no procedural complications, leading to same-day discharge. The other 2 patients faced complications: one experienced an LAA perforation, requiring a conversion from DS to GA for surgical intervention; the second developed a pericardial effusion during the procedure, though without the need for anesthesia conversion or surgical intervention.

One of our patients experienced a laceration of the RV; this occurred after the observation of pericardial tamponade after the successful implantation of the closure device, leading to the placement of pericardial drains. After draining approximately half a liter of blood, the patient was urgently transferred to the operating room, where the atrial appendage had a hematoma but remained intact, and a small RV perforation was identified and repaired.

We consider that the placement of the drains may have iatrogenically caused the perforation, and the initial effusion, which later developed into tamponade, may have originated from a small atrial perforation that had spontaneously sealed by the time of the surgical procedure. Despite this significant complication, the patient survived, the device remained implanted, and no other complications were found during the hospitalization or 45-day TEE and follow-up. None of our patients died of any procedure-related complications.

Most of our patients, 145 (97%), were discharged on the same day as the procedure. Nonetheless, there were 5 exceptions: 2 patients had to stay longer for social reasons unrelated to the procedure and the remaining 3 for facing complications during the procedure.

During the 45-day follow-up visit, 146 (98%) underwent follow-up imaging. The remaining 4 patients did not get follow-up imaging because of different reasons. One of the patients did not have insurance at the time, while the other 3 were the ones who did not receive the implanted device. A single patient experienced laryngospasms during TEE probe insertion at the 45-day follow-up visit; as a result, a cardiac computed tomography scan was performed instead.

None of our patients experienced adverse reactions to the anesthesia. In addition, 141 (94%) did not require anesthesia conversion. Only 9 patients (6%) required anesthesia conversion; 7 of 9 were due to unforeseen factors, such as laryngospasms or difficulty passing the TEE probe, while only 2 were due to procedural complications.

In a comparable study by Ates et al,^12^ 112 patients with elevated CHA_2_DS_2_-VASc scores (a median of 3 points) underwent TEE-guided LAAC between November 2018 and November 2019 using a similar anesthetic protocol named moderate conscious sedation (MCS). They reported no complications from the anesthesia, which is consistent with our results. Notably, in their study, no anesthesia conversions were required. It is pertinent to mention that the closure device they used was a previous version of the WATCHMAN FLX device, known as the WATCHMAN device (Boston Scientific).

In another study by the same group of authors, Marmagkiolis et al^13^ reported that 112 additional patients with elevated CHA_2_DS_2_-VASc scores (a median of 3 points) underwent TEE-guided LAAC using the WATCHMAN device under MCS between August 2019 and May 2020. The authors observed no complications from MCS this time, and none of their patients required conversion. These results also align closely with the findings we reported herein.

Some authors, such as Golzarian et al,^14^ have introduced variations to the traditional 2-operator approach, as demonstrated in their SOLO-CLOSE method. In SOLO-CLOSE, a single operator conducts LAAC under TEE guidance using a nurse-driven conscious sedation protocol. This strategy eliminates the need for preprocedural imaging and reduces the required specialists for LAAC to just one.

In our approach, the need for a permanent anesthesiologist was eliminated by incorporating a certified registered nurse anesthetist capable of administering anesthesia throughout the entire procedure, with an anesthesiologist ready to step in if complications arise. Implementing the DS protocol also reduces recovery times and minimizes overnight hospital stays. In addition, we simplified the process by conducting screening TEE on the same day as LAAC, removing the need for separate scheduling of preprocedural imaging.

We also suppressed the necessity for a noninvasive cardiologist by using the assistance of a certified echocardiography technician, which may further reduce the overall costs. Moreover, our approach contributes to a reduction in in-hospital time by facilitating same-day patient discharge.

The limitations of our study include the absence of a comparative group for outcome assessment. While our study demonstrated excellent outcomes with few complications, the lack of a comparison group hinders a direct assessment of the efficacy of our approach. This limitation arises from the predominant use of DS for almost all LAAC procedures conducted in our center, a method that has been consistently successful for at least a couple of years.

Additional limitations are that including patients from a single center may restrict the external validity of our findings. Moreover, the lack of random sampling introduces a potential selection bias. Despite the study being conducted in a single center, which may limit generalizability, the substantial sample size enhances the statistical robustness of our results. Finally, using a single closure device (WATCHMAN FLX) may restrict the generalizability of our findings compared with outcomes associated with other devices.

On the basis of our study findings and evidence from other authors, we believe that our approach is safe and feasible, offering comparable outcomes and minimal complications. Moreover, DS could be a safe alternative to GA. This could lead to cost-effectiveness and improved patient outcomes.

We advocate for the adoption of protocols that facilitate the availability of LAACs, incorporating same-day screening imaging and discharge, particularly in centers with a limited number of specialists. As demonstrated, a properly trained technician can safely replace the role of a noninvasive cardiologist.

We believe that our study contributes valuable evidence supporting the feasibility and safety of our approach, emphasizing the role of DS as a standardized alternative to GA in the performance of LAACs.

## Conclusion

Our novel single-operator-technician approach under DS is safe, feasible, and potentially cost-effective. Moreover, DS could be a safe alternative to GA when performing LAACs. Adopting protocols that facilitate the availability of LAACs, incorporating same-day screening imaging and same-day discharge, particularly in centers with a limited number of specialists, could help make LAACs more widely available and potentially reduce procedural costs.

## Disclosures

While the authors did not receive specific funding or sponsorship for this manuscript's publication, Drs Stone, Abdelrahim, Crossen, and Prasad were compensated for performing percutaneous left atrial appendage closures as electrophysiologists. The remaining authors declared no conflicts of interest.
